# Usefulness of Point-Of-Care Ultrasound in Diagnosing and Managing Pediatric Multidistrict Chylous Effusion

**DOI:** 10.3390/reports7040110

**Published:** 2024-12-05

**Authors:** Tommaso Bellini, Marta Bustaffa, Marco Crocco, Federica Casabona, Giorgia Iovinella, Federica Malerba, Matteo D’Alessandro, Emanuela Piccotti

**Affiliations:** 1 Pediatric Emergency Room and Emergency Medicine Unit, Emergency Department, IRCCS Istituto Giannina Gaslini, 16147 Genoa, Italy; martabustaffa@gaslini.org (M.B.); matteodalessandro@gaslini.org (M.D.); emanuelapiccotti@gaslini.org (E.P.); 2 Pediatric Gastroenterology and Endoscopy Unit, IRCCS Istituto Giannina Gaslini, 16147 Genoa, Italy; marcocrocco@gaslini.org; 3 Department of Neuroscience, Rehabilitation, Ophthalmology, Genetics, Maternal and Child Health (DINOGMI), University of Genoa, 16147 Genoa, Italy; 3907391@studenti.unige.it (F.C.); 3672473@studenti.unige.it (G.I.); federicamalerba@gaslini.org (F.M.)

**Keywords:** chylothorax, coeliac disease, lymphangiodysplasia, pediatric emergency department, pleural effusion, POCUS

## Abstract

**Background and Clinical Significance**: The use of point-of-care ultrasound (POCUS) in emergency departments is rapidly growing due to its ability to provide immediate and accurate diagnostic information at the bedside. Furthermore, it can provide precise and rapid information on the location of multidistrict effusions in patients with suspected lymphatic decompensation. **Case Presentation**: This unique clinical case report describes a patient who presented with massive, multidistrict chylous effusion secondary to acute lymphatic insufficiency, a rare and challenging condition. Due to a recent diagnosis of celiac disease, the patient had started a gluten-free diet ten days before the onset of symptoms, suggesting a possible causal link. Through comprehensive thoracoabdominal POCUS, the diagnosis was made promptly, avoiding delays in treatment and enabling timely decision-making. **Conclusions**: This case emphasizes the critical role of POCUS not only in expediting diagnosis but also in guiding invasive procedures, such as thoracentesis, by visualizing fluid accumulation and anatomical structures in real-time. Moreover, POCUS provides an invaluable tool for ongoing clinical ultrasound follow-up, facilitating continuous monitoring without exposing the patient to the risks of radiation, thus optimizing patient care and resource utilization.

## 1. Introduction and Clinical Significance

Chylothorax, characterized by the accumulation of chyle in the pleural space, is often caused by disruption of the thoracic duct [1,2]. However, its precise incidence in pediatric populations remains uncertain, and its etiology is often unknown [1,2]. The primary causes of chylothorax in pediatric populations are congenital lymphatic malformations; surgical trauma, especially after cardiothoracic procedures; malignancies; and systemic conditions such as infections or syndromic disorders like Noonan syndrome or Down syndrome [1,2,3].

Chylothorax may be particularly concerning when it is massive as it can extend beyond the pleural space and involve the pericardial and peritoneal cavities, leading to serious complications. Although rare in most children, it is the leading cause of pleural effusion in neonates [1]. When undiagnosed, chylothorax can lead to life-threatening acute respiratory distress and significant nutritional deficiencies as it is associated with protein loss [1]. Therefore, timely diagnosis and management are crucial to prevent complications. Point-of-care ultrasonography (POCUS) is a valuable tool in this context, providing a rapid, non-invasive, and repeatable imaging modality allowing clinicians to promptly identify pleural effusions at the bedside, aiding in the early diagnosis and management of chylothorax in children [3,4,5,6,7].

We present a unique clinical case of a patient with massive multidistrict chylous effusion due to acute lymphatic failure with impaired lipid absorption in which POCUS allowed for prompt clinical management.

## 2. Case Presentation

This case refers to a previously healthy 11-year-old girl who was transferred to the Gaslini third-level Pediatric Emergency Department (PED) from another regional spoke hospital. She was admitted because of low-grade fever, asthenia, mild respiratory distress, and a rash on the lower extremities. Ten days before admission, the patient started a gluten-free diet due to a diagnosis of celiac disease. Upon admission, she presented in good clinical condition, afebrile, slightly tachypneic, with a respiratory rate of 32 breaths per minute, and normal SaO2. The remaining vital parameters were within the normal limits for her age, and the blood test results were negative or unremarkable. 

**Figure 1:**
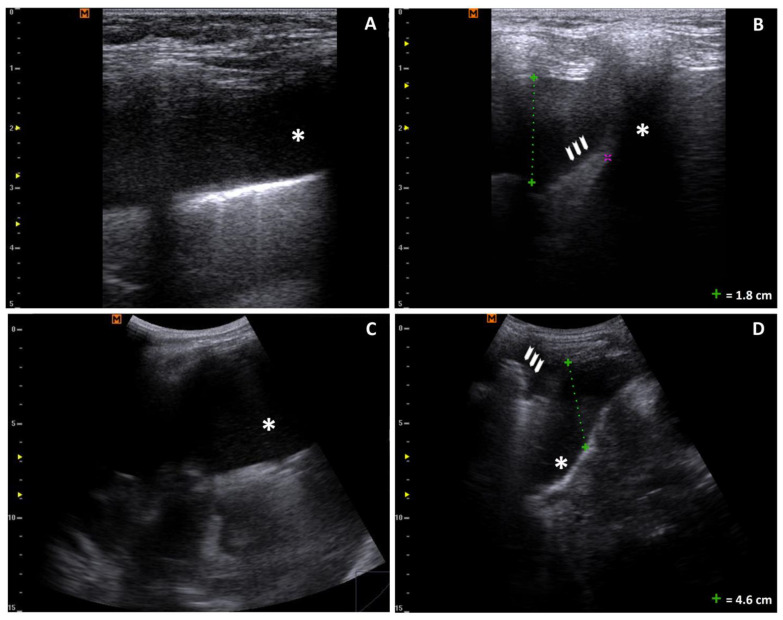
Bilateral pleural effusion: (**A**,**B**) longitudinal scan of the right lung with a linear probe showing a 1.8 cm anechoic pleural effusion (*) and underlying atelectasic lung (arrowheads); the purple cursor is a measurement typo; (**C**,**D**) longitudinal scan with a convex probe showing massive anechoic pleural effusion (*) and underlying atelectasic lung (arrowheads).

**Figure 2:**
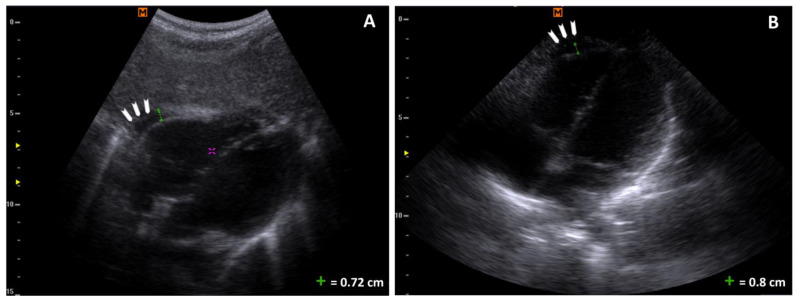
Pericardic effusion: (**A**) a four-chamber subcostal scan with an effusion of maximum thickness of 0.78 cm (arrowheads); the purple cursor is a measurement typo; (**B**) a four-chamber apical scan with an effusion of maximum thickness of 0.8 cm (arrowheads).

**Figure 3:**
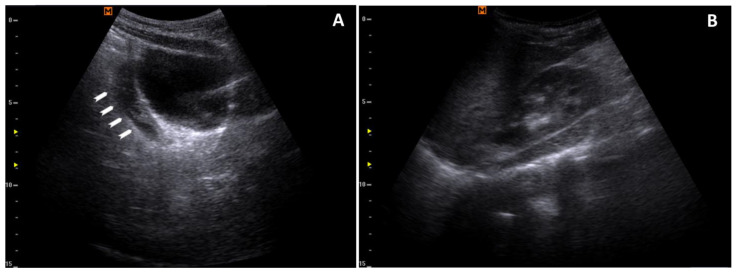
Peritoneal effusion: (**A**) a longitudinal scan of the bladder with retrovesical effusion (arrowheads); (**B**) the absence of effusion in the hepatorenal recess.

Upon admission to our PED, a complete POCUS was performed, which showed bilateral pleural effusion, mild pericardial effusion, and ascites without other signs of pulmonary parenchymal or cardiac involvement (Figure 1, Figure 2 and Figure 3). Bedside focused cardiac ultrasonography (FoCUS) allowed us to exclude systolic dysfunction or cardiac tamponade.

Ultrasound-guided thoracentesis was performed based on the results of POCUS. Chemical–physical examination of the pleural fluid confirmed the diagnosis of chylothorax [1]. Table 1 reports the result of pleural effusion examination.

The finding of a chylous multidistrict effusion prompted further diagnostic investigations. A chest CT scan, which was performed to exclude possible lymphoma, was negative. Moreover, lymphoscintigraphy and lymphangio-magnetic resonance (MRI) were unremarkable. She was started on a fat-free diet with daily ultrasound of pleural, pericardial, and peritoneal effusions. The patient showed progressive improvement until the effusions were reabsorbed, and she was discharged 12 days after hospitalization. Therefore, the patient was fed a gluten- and lipid-free diet.

Clinical and ultrasound evaluations were performed daily for the duration of hospitalization then weekly for a month and monthly for the following six months to evaluate any relapses during the reintroduction of lipids into the diet.

After two months, the lipids were progressively reintroduced, and at six months of follow-up, there were no clinical relapses.

## 3. Discussion

To our knowledge, this is the first reported case of lymphatic insufficiency with multidistrict chylous effusions secondary to the initiation of a gluten-free diet. Moreover, this report aimed to demonstrate the diagnostic and therapeutic role of POCUS in managing multidistrict chylous effusions in children, as well as the rarity of this condition in pediatric patients.

As stated before, the described case refers to a chylothorax, probably formed due to an inappropriate fat-rich diet, leading to an imbalance in lymphatic function [1,2]. Lymphatic effusion findings are consistent with negative inflammatory markers and the absence of possible inflammatory or infectious etiologies. The execution of POCUS upon admission to the PED, highlighting the presence of significant pleural, pericardial, and peritoneal effusions without clear pulmonary and cardiac involvement, immediately addressed the diagnostic suspicion of lymphatic involvement [3,8,9]. 

Early ultrasound evaluation together with ultrasound-guided thoracentesis immediately confirmed the diagnosis, and examination of the pleural fluid confirmed the presence of chyle [4,10]. The immediate use of POCUS significantly accelerates the diagnostic time, avoiding the maximum use of radiological tests, which would probably not have provided further useful elements [4,5,11]. Moreover, FoCUS excludes systolic dysfunction or cardiac tamponade [3,4,5,6,10,11].

A chest CT examination was subsequently performed to exclude mediastinal obstructive causes, the most probable being lymphoma, other malformative causes such as thoracic duct abnormalities, and superior vena cava compression, which could have interfered with normal lymphatic drainage and may have required surgical management [12,13]. The patient subsequently underwent specific tests to diagnose lymphatic dysplasia.

Moreover, inflammatory markers were persistently negative, allowing us to exclude infectious and autoimmune causes. The only pathological test found was fecal alpha-1 antitrypsin (α1AT), suggesting intestinal protein-losing enteropathy, probably secondary to uncontrolled celiac disease. However, serum albumin levels were normal, indicating an acute episode [8,14].

In addition, lymphoscintigraphy and lymphangio-MRI were negative, allowing us to exclude both functional and anatomical involvement of lymphatic vessels or lymphangiodysplasia [1,2,8,12].

These exams clearly showed no malformative involvement of the lymphatic system, suggesting that the cause could be an acute imbalance related to massive intestinal lymph production. We can speculate that the large quantity of fat ingested with the gluten-free diet may have led to the inability of the system to dispose of this excess lymph through the thoracic duct, producing a reflux of lymph at the pleural, pericardial, and peritoneal levels. The lack of signs of pulmonary and cardiac involvement allowed us to exclude the presence of pulmonary lymphangiectasia, which is similar to the absence of intestinal lymphangiectasia [3,4,6,13]. To date, we have not found any previous reports in the current medical literature linking massive chylous effusion to a hyperlipidic diet.

Chylopericardium is an unusual and rare clinical entity that is rarely isolated [15]. It can result from recent cardiac or thoracic surgery, trauma, mediastinal tumors, or central venous hypertension, although most cases are idiopathic with no clear mechanical obstruction of the thoracic duct. Diagnosis is typically confirmed via pericardiocentesis, which reveals milky fluid with high triglyceride levels [15]. In this case, given the exclusion of all the above-mentioned etiologies, we opted for a conservative approach by analyzing the pleural fluid, which was more accessible, and inferred the chylous nature of the cardiac effusion, confirmed by its resolution after starting a fat-free diet.

As stated before, this is the first case report of a diagnosis of multi-district, pleural, pericardial, and peritoneal effusion due to lymphatic system failure using POCUS [4,5,11]. Furthermore, this is the first described case highlighting a lymphatic failure secondary to the initiation of a gluten-free diet, although our study remains a diagnostic hypothesis. Notwithstanding this, the clinician should be alarmed by acute respiratory failure in a patient recently placed on a gluten-free diet because it may be an expression of lymphatic decompensation.

Anechoic effusions did not allow reliable differentiation between transudative and exudative fluid [5,11]. However, the bilateral presence of pleural effusion without an underlying pulmonary pathology, pericardial effusion with normal cardiac function, and ascitic effusion allowed us to assume a transudative nature [4,5,6,11].

In addition to confirming clinical suspicion at the time of admission, POCUS allowed thoracentesis to be performed quickly, and the diagnosis was confirmed. Furthermore, we employed POCUS and accurate daily clinical ultrasound follow-up until effusions were completely resolved. An early diagnosis is of fundamental importance, allowing us to choose the necessary further tests and the ideal treatment for the patient based on diagnostic suspicion. Finally, serial ultrasound examinations allowed evaluation of the efficacy of a lipid-free diet before considering any further therapies, such as octreotide, a synthetic somatostatin analog that may reduce the production of lymph and its leakage into the pleural space [2,3,7,11].

## 4. Conclusions

This clinical case highlights a newly described entity, namely, the potential onset of massive chylous effusion secondary to acute lymphatic insufficiency caused by a hyperlipidic gluten-free diet. Therefore, patients experiencing respiratory distress after the initiation of a gluten-free diet should be evaluated for suspected chylous pleural effusion. Moreover, this report support the pivotal role of POCUS in the rapid and accurate diagnosis of multidistrict chylous effusion in a pediatric patient. The early use of POCUS allowed for the timely identification of pleural, pericardial, and peritoneal effusions, leading to prompt clinical management and the avoidance of serious complications. Finally, POCUS enabled bedside invasive procedures, such as ultrasound-guided thoracentesis, to be performed with precision and minimal delay.

## Figures and Tables

**Table 1:** Diagnostics values for chilotorax and patient’s results.

Exam	Chilotorax Values	Result
Cells (el./mm^3^)	300–400	335, prevalent lymphocytes
Aerobic culture	Negative	Negative
Anaerobic culture	Negative	Negative
Triglycerides (mg/dL)	>50	2465
Total cholesterol (mg/dL)	60–200	114
Total proteins (g/dL)	3–5	3.97

## Data Availability

The data presented in this study are available on request from the corresponding author due to privacy.
